# The Levels of Oxidized Phospholipids in High-Density Lipoprotein During the Course of Sepsis and Their Prognostic Value

**DOI:** 10.3389/fimmu.2022.893929

**Published:** 2022-05-03

**Authors:** Zhaohong Li, Zengtao Luo, Xiaoqian Shi, Baosen Pang, Yingmin Ma, Jiawei Jin

**Affiliations:** ^1^Department of Respiratory and Critical Care Medicine, Beijing Institute of Respiratory Medicine, Beijing Chaoyang Hospital, Capital Medical University, Beijing, China; ^2^The Clinical Research Center, Beijing Chaoyang Hospital, Capital Medical University, Beijing, China; ^3^Department of Respiratory and Critical Care Medicine, Beijing Youan Hospital, Capital Medical University, Beijing, China

**Keywords:** sepsis, high-density lipoprotein (HDL), oxidized phospholipids, 1-palmitoyl-2-(5-oxovaleroyl)-sn-glycero-phosphatidylcholine (POVPC), 1-palmitoyl-2glutaroyl-sn-glycero-phosphatidylcholine (PGPC)

## Abstract

**Purpose:**

To examine the levels of 1-palmitoyl-2-(5-oxovaleroyl)-sn-glycero phosphatidylcholine (POVPC) and 1-palmitoyl-2-glutaroyl-sn-glycero-phosphatidylcholine (PGPC) (the oxidized phosphatidylcholines) in HDL during the course of sepsis and to evaluate their prognostic value.

**Materials and Methods:**

This prospective cohort pilot study enrolled 25 septic patients and 10 healthy subjects from 2020 to 2021. The HDLs were extracted from patient plasmas at day 1, 3 and 7 after sepsis onset and from healthy plasmas (total 81 plasma samples). These HDLs were then subjected to examining POVPC and PGPC by using an ultra-high performance liquid chromatography coupled with tandem mass spectrometry (UHPLC-MS/MS) system. We further measured the levels of 38 plasma cytokines by Luminex and evaluated the correlation of HDL-POVPC level with these cytokines. Patients were further stratified into survivors and non-survivors to analyze the association of HDL-POVPC level with 28-day mortality.

**Results:**

Septic patients exhibited significant increase of HDL-POVPC at day 1, 3 and 7 after sepsis onset (POVPC-D1, p=0.0004; POVPC-D3, p=0.033; POVPC-D7, p=0.004, versus controls). HDL-PGPC was detected only in some septic patients (10 of 25) but not in healthy controls. Septic patients showed a significant change of the plasma cytokines profile. The correlation assay showed that IL-15 and IL-18 levels were positively correlated with HDL-POVPC level, while the macrophage-derived chemokine (MDC) level was negatively correlated with HDL-POVPC level. Furthermore, HDL-POVPC level in non-survivors was significantly increased versus survivors at day 1 and 3 (POVPC-D1, p=0.002; POVPC-D3, p=0.003). Area under ROC curves of POVPC-D1 and POVPC-D3 in predicting 28-day mortality were 0.828 and 0.851. POVPC-D1and POVPC-D3 were the independent risk factors for the death of septic patients (p=0.046 and 0.035).

**Conclusions:**

HDL-POVPC was persistently increased in the course of sepsis. POVPC-D1 and POVPC-D3 were significantly correlated with 28-mortality and might be valuable to predict poor prognosis.

## Introduction

Sepsis, represented as the dysregulated systemic inflammation syndrome in response to infection, is a common pathway to death in infectious diseases. The tissue injuries and the organ failures during sepsis are involved with complex interactions between microbes, immune cells, coagulation cascade, and endothelium (1, 2). Some clinical treatments, including appropriate antibiotic administration and fluid resuscitation, are suggested to improve outcomes of septic patients, but sepsis remains a high mortality rate (3). The reliable diagnostic and prognostic biomarkers are critical for constraining its mortality through guiding precise and effective therapeutic strategies.

Some clinical markers, such as procalcitonin (PCT) and C-reactive protein (CRP), have predictive value for septic prognosis, but their specificity or sensitivity are significantly limited by the clinical and pathogenetic heterogeneities of sepsis (4). Of note, the increasing lipidomic studies indicate that lipid metabolism remodeling plays roles in septic pathophysiology (5). Given that most alterations in cellular function, inflammatory response and energy imbalance during sepsis could lead to the changes in lipid metabolism, the lipids biomarkers show advantages in prognosis of septic patients (6).

High-density lipoprotein (HDL), as a critical mediator in lipid metabolic homeostasis, has notable anti-inflammatory and anti-oxidation roles in septic response (7). The decrease in plasma level of HDL-cholesterol (HDL-C) is correlated with a poor prognosis in septic patients (8–11). Notably, recent studies indicate an adverse transition of HDL to pro-inflammatory properties, in acute inflammatory disorder diseases including sepsis (8, 12, 13). Our previous study further demonstrates that the HDL from patients with sepsis-induced ARDS significantly promotes endothelial barrier dysfunction, which is associated with the remodeling in HDL constitutes, such as the increases of apoC-III, apoE, and serum amyloid A (SAA) (14). We hypothesized that the constitute changes in apoproteins would cause the alterations in lipid constitute and such lipid remodeling might be involved in adverse HDL transition.

Oxidized phospholipids (Ox-PLs) have significant pro-inflammatory function contributing to the pathogenesis of acute and chronic infection diseases (15). 1-palmitoyl-2-arachidonoyl-sn-glycero-3-phosphatidylcholine (PAPC) is located in lipoproteins and it is oxidatively truncated in the sn-2 fatty acid residues to oxidized PAPC (Ox-PAPCs), including 1-palmitoyl-2-(5-oxovaleroyl)-sn-glycero-phosphatidylcholine (POVPC) and 1-palmitoyl-2glutaroyl-sn-glycero-phosphatidylcholine (PGPC) (16). The ox-PAPCs referenced as active principles of Ox-LDL have been involved in pro-inflammatory effects on the endothelial cells and innate immune cells in the pathogenesis of atherosclerosis and acute lung injury (17). Intriguingly, HDL plays a critical role to prevent Ox-LDL formation due to its anti-oxidation property (18). It has been shown that phospholipids in HDL itself are more susceptible to oxidation (19). However, no study has yet examined the changes in the levels of POVPC and PGPC in HDL during inflammatory response such as sepsis.

Herein, we examined the levels of POVPC and PGPC in plasma HDL from healthy controls and the septic patients at Day 1, 3, and 7 from septic diagnosis. We further conducted the correlation analysis of the level of HDL POVPC to clinical outcome. Our results could shed additional light on developing an early-stage biomarker for septic prognosis.

## Material and Methods

### Study Design and Participants

This study was a prospective cohort pilot study that was conducted at the Department of Pulmonary and Critical Care Medicine and at the emergency department in Beijing Chao-Yang Hospital. All enrolled adult patients with sepsis (ages ≥ 18 years) met the Sepsis-3 criteria: an acute elevation of sequential organ failure assessment (SOFA) scores ≥ 2 points upon suspected or confirmed infections (20). The patients were excluded as follows: patients died within 3 days; patients with diabetes mellitus or hyperthyroidism; patients diagnosed with hypercholesterolemia; patients who received statin drugs; patients who were pregnant. There were 13 patients further excluded because their blood samples were not collected at Day 3 due to clinical conditions. Finally, a total of 25 eligible septic patients and 10 of sex- and age-matched healthy subjects were included, in which 24 of 25 septic patients were infected with bacteria, and only 1 patient was infected with fungi. All the study participants provided informed consent for the study. Our protocol and procedures were approved by the Ethics Committee of Beijing Chao-Yang Hospital (approval No.: 2021-ke-313).

### Primary Endpoints

The primary endpoint of interest was the 28-day mortality from diagnosis and the goal was to explore the correlation of HDL POVPC level to the 28-day mortality in these enrolled septic patients. The enter logistic regression was performed to identify the independent risk factors for the 28-day mortality of sepsis.

### Clinical Data Collection

Demographic characteristics, vital signs and comorbidities at admission, laboratory data, SOFA score, acute physiology, and chronic health evaluation II (APACHE II) score at septic diagnosis, treatments during RICU, including vasopressors administration and continuous renal replacement therapy (CRRT) and survival information were collected.

### Blood Collection and Cytokines Detection

To track changes of POVPC and PGPC during their pathophysiological processes, whole blood was collected at Day 1 (the day at diagnosis), Day 3, and Day 7 from septic diagnosis as well as from healthy controls at enrollment. At Day 7 of sepsis, only 21 samples were collected due to the death of 4 patients within 7 days. Finally, a total of 81 whole blood samples was collected in the 5-mL EDTA blood collection tubes. The plasmas were obtained after centrifugation at 1800 g for 15 min and immediately stored at -80°C until use. The EDTA plasmas were further subjected into an HDL isolation procedure and the multiplex inflammatory cytokines assays using the 38-cytokine panel MILLIPLEX^®^ MAP (Merck Millipore, HCYTA-60K-PX38).

### Isolation of High-Density Lipoprotein

A 500 μL of plasma from each subject was used in the procedure of HDL isolation as previously described (21). Briefly, after pre-staining lipoproteins using Sudan 7b to facilitate visualization, the very low-density lipoprotein (VLDL), low-density lipoprotein (LDL), and HDL fractions were separated using a sequential procedure according to their density differences (HDL, ρ = 1.478 g/ml; LDL, ρ = 1.182 g/ml; VLDL, ρ = 1.006 g/ml). The solutions with different densities adjusted by dissolving different qualities of NaBr and NaCl in MilliQ water, were successively layered on serum samples. The layers were centrifuged at 180,000 g with p65a rotor on cp80wx ultracentrifuge (Hitachi) for 12 h to remove VLDL and LDL, and collected purified HDL finally. The purity of HDL was confirmed by Western blot analysis of apoprotein A-1 (apoA-1) and apoprotein B 100 (apoB100) (**Supplementary Figure S1**).

### Targeted Metabolomics

The POVPC and PGPC lipid standards and the stable isotope-labeled standards were obtained from Sigma-Aldrich (St. Louis, MO). Briefly, the 10 μL of sample was added to 490 μL Calibrator Diluent followed by well vortexed and then 50 μL of the dilution was homogenized with 200 μL of acetonitrile/methanol (1:1) containing internal standards. After centrifuge at 12,000 rpm for 10 min, the supernatant was injected into the LC-MS/MS system. An ultra-high performance liquid chromatography coupled with tandem mass spectrometry (UHPLC-MS/MS) system (UHPLC-QTRAP 6500^+^) was used to quantitate lipids (**Supplementary Figure S2** and **Supplementary Table S1**). The standard curves for POVPC and PGPC were built by the concentration series of standard solution (**Supplementary Figure S3**).

### Statistical Analysis

Continuous and categorical variables are expressed as interquartile ranges (IQRs) and as numbers and percentages, respectively. For the comparisons between two groups, the Student’s t-test or Mann-Whitney U test was performed for continuous variables, and the Fisher’s exact test was performed for categorical variables. For the comparisons of three groups, the one-way ANOVA (Bonferroni *post hoc* test) was performed for normally distributed variables and one-way Kruskal–Wallis tests (Dunn’s *post hoc* test) was performed for non-normally distributed variables. Receiver operating characteristic (ROC) curve was performed for the prediction of 28-day mortality with POVPC, and also compare the sensitivity and specificity with SOFA, C-reactive protein, and lactate; the cutoff value was selected based on the highest Youden’s index in order to maximize both sensitivity and specificity (Youden’s index=sensitivity+specificity-1). The correlation analysis was performed with the Pearson method. Furthermore, enter logistic regression was performed to identify the risk factors for the 28-day mortality (factors with p < 0.05 in univariate logistic regression were in turn subjected to multivariate logistic regression). A 2-sided *P* < 0.05 was considered statistically significant. SPSS version 24.0 (SPSS, Chicago, IL) statistical software was used in this study.

## Results

### Comparison Between Septic Patients and Healthy Controls

**Figure 1:**
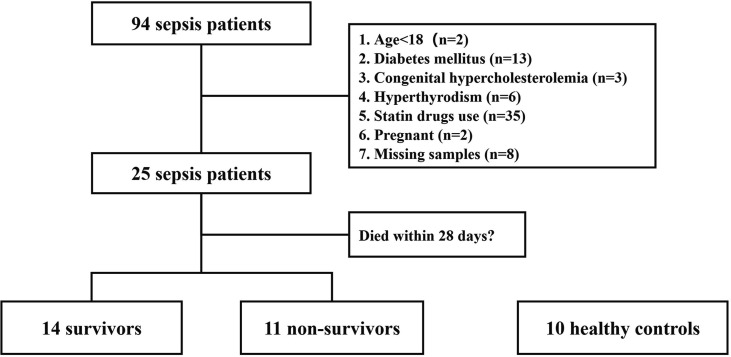
Flowchart of patient enrollment. This is a study flow chart of septic patients along with healthy controls.

A total of 25 septic patients and 10 healthy controls were included as indicated by the subject enrollment flowchart (**Figure 1**). There were 80% of the septic patients with pulmonary infection (20/25) and 20% of patients with abdominal infection (5/25). Moreover, most of the patients (24/25, 96%) had bacterial infection, and only 1 patient had fungal infection. The characteristics of recruited subjects at diagnosis are shown in **Table 1**. No significant differences between the two groups, in age, sex, and body mass index (BMI), were observed. In laboratory data, septic patients showed significant decreases in the levels of total plasma cholesterol (CHOL, 3.0 vs. 4.7 mmol/L, p = 0.0001), HDL cholesterol (HDL-C, 0.9 vs. 1.5 mmol/L, p = 0.001), and LDL cholesterol (LDL-C, 1.6 vs. 2.7 mmol/L, p = 0.008).

**Table 1:** Characteristics of septic patients and healthy controls.

Variables	Septic patients (n=25)	Healthy controls (n=10)	P value
**Age (years)**	71 (59~82.5)	64.0 (62.8~69)	0.411
**Men, n (%)**	17 (68)	6 (60)	0.706
**Body mass index (kg/cm²)**	23.5 (21.6~25.4)	24.2 (20.6~25.5)	0.959
**Total cholesterol (mmol/L)**	3.0 (1.8~4.1)	4.7 (4.3~5.6)	**0.0001**
**HDL-C (mmol/L)**	0.9 (0.6~1.2)	1.5 (1.1~1.8)	**0.001**
**LDL-C (mmol/L)**	1.6 (0.8~2.4)	2.7 (2.0~3.2)	**0.008**
**Targeted metabolic analysis**			
**POVPC-D1 (ng/µL)**	292.0 (254.7~456.0)	224.9 (210.3~239.8)	**0.0004**^&^
**POVPC-D3 (ng/µL)**	313.7 (232.6~505.7)	—	**0.033**^&^
**POVPC-D7 (ng/µL)**^▲^	288.7 (209.5~458.7)	—	**0.004**^&^
**PGPC-D1, detected, n (%)**	5 (20.0)	ND	
**PGPC-D3, detected, n (%)**	6 (24.0)	ND	
**PGPC-D7, detected, n (%)**^▲^	4 (19.0)	ND	

HDL-C, high-density lipoprotein cholesterol; LDL-C, low-density lipoprotein cholesterol; POVPC-D1/D3/D7, the 1-palmitoyl-2-(5-oxovaleroyl)-sn-glycero-phosphatidylcholine levels in HDL at Day 1 or Day 3 or Day 7; PGPC-D1/D3/D7: the detected numbers of subjects of 1-palmitoyl-2-glutaroyl-sn-glycero-phosphatidylcholine at Day 1 or Day 3 or Day 7; ND, not detected. &, compared to healthy controls. ▲, a total of 21 plasma samples were collected due to the death of 4 patients at Day 7. p value <0.05 was in bold.

**Figure 2:**
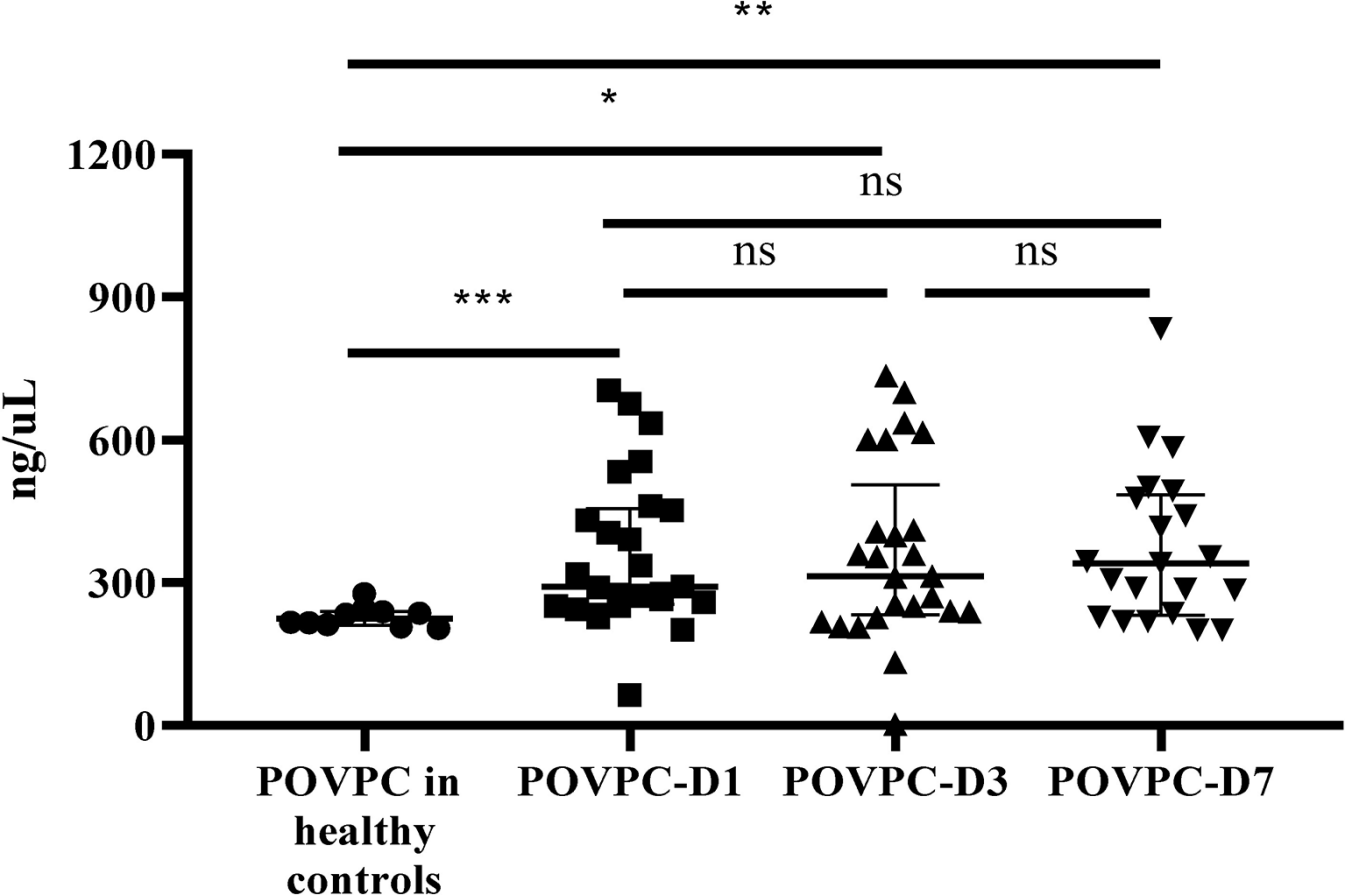
The persistent increase of HDL POVPC in septic patients vs. healthy controls. The difference in HDL POVPC between healthy controls and septic patients, and the increased levels were persistent in the course of sepsis. Data were presented in median with interquartile range. N=10 for healthy controls, n=14 for survivors, n=11 for non-survivors (D1 and D3), n=7 for non-survivors (D7); POVPC, 1-palmitoyl-2- (5-oxovaleroyl) -sn-glycero-phosphatidylcholine; D1, Day 1; D3, Day 3; D7, Day 7. *p<0.05; **p<0.01; ***p<0.001; ns, not significant.

POVPC level in HDL from septic patients was significantly increased at Day 1 (POVPC-D1), Day 3 (POVPC-D3), and Day 7 (POVPC-D7) after septic onset (292.0 vs. 224.9 ng/μL, p = 0.0004; 313.7 vs. 224.9 ng/μL, p = 0.033; 288.7 vs. 224.9 ng/μL, p = 0.004, respectively) (**Table 1** and **Supplementary Tables S2, S3**). We also examined the PGPC in HDL. PGPC was not detected in HDL from all healthy controls, and it was only detected in HDL from part of septic patients (10 of 25, **Table 1** and **Supplementary Tables S2, S3**). Furthermore, there was no significant difference among POVPC-D1, POVPC-D3, and POVPC-D7 of all septic patients (p > 0.99 for all these three groups) (**Figure 2**).

### The Comparison Between Septic Survivors and Non-Survivors

**Figure 3:**
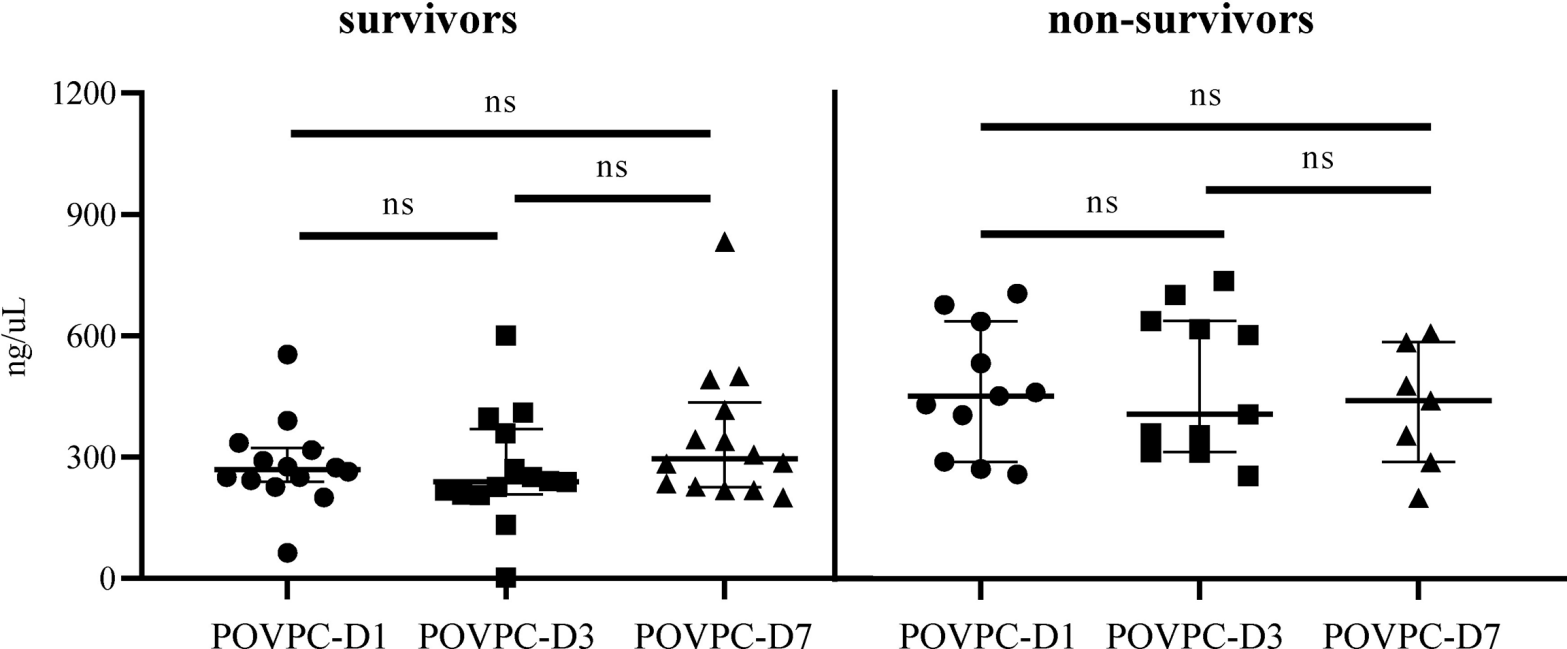
The difference in HDL POVPC of septic survivors and non-survivors at different time points. The difference in HDL POVPC of septic survivors and non-survivors at Day 1, Day 3, and Day 7. POVPC, 1-palmitoyl-2- (5-oxovaleroyl)-sn-glycero-phosphatidylcholine; D1, Day 1; D3, Day 3; D7, Day 7; ns, not significant.

Patients were subgrouped into survivors and non-survivors according to the 28-day mortality (the primary outcome). The comparisons of clinical characteristics between two groups are shown in **Table 2**. Non-survivors exhibited higher levels of C reactive protein (CRP), serum lactate, and SOFA score (210.9 vs. 119.1 mg/L, p = 0.025; 5.2 vs. 2.1 mmol/L, p = 0.048; 11 vs. 7.5, p = 0.015, respectively). Non-survivors also displayed a decrease in the level of plasma HDL-C vs. survivors, although the difference failed to achieve the statistical significance (0.8 vs. 1.0 mmol/L, p = 0.067). Of note, non-survivors showed a significantly increased level of POVPC in HDL at Day 1 and Day 3 after septic onset (POVPC-D1: 451 vs. 270 ng/μL, p = 0.002; POVPC-D3: 406.4 vs. 239.5 ng/μL, p = 0.003). There were no significant differences between the septic survivors and non-survivors in ventilator-free days, ICU days, and the complications and treatments (**Table 3**). Moreover, there is also no significant difference among POVPC-D1, POVPC-D3, and POVPC-D7 in septic survivors or non-survivors (survivors: POVPC-D1 vs. D3, p > 0.99; POVPC-D1 vs. D7, p > 0.99; POVPC-D3 vs. D7, p = 0.46; non-survivors: p > 0.99 for all these three groups) (**Figure 3**).

**Table 2:** Characteristics of septic survivors and septic non-survivors.

Variables	Total (n=25)	Sepsis (n=25)		
		Survivors (n=14)	Non-survivors (n=11)	P value
**Age (years)**	71 (59~82.5)	64 (54~79.8)	76 (67~84)	0.051
**Men, n (%)**	17 (68)	9 (64.3)	8 (72.7)	0.496
**Body mass index (kg/cm²)**	23.5 (21.6~25.4)	23.7 (21.9~26.5)	23.3 (19.3~23.7)	0.311
**Etiology, n (%)**				
**Pneumonia**	20 (80.0)	11 (78.6)	9 (81.8)	1
**Abdominal infection**	5 (20.0)	3 (21.4)	2 (18.2)	1
**Underlying diseases, n (%)**				
**Hypertension**	4 (32.0)	4 (28.6)	4 (36.4)	0.648
**Cardiovascular disease**	12 (48.0)	5 (35.7)	7 (63.6)	0.238
**Chronic kidney disease**	5 (20.0)	4 (28.6)	1 (9.1)	0.341
**Malignancy**	5 (20.0)	2 (14.3)	3 (27.3)	0.623
**Laboratory data at day 1**				
White blood cell (*10^9/L)	11.1 (8.4~17.9)	11.0 (8.5~13.9)	15.9 (7.3~18.7)	0.352
NE (*10^9/L)	10.0 (7.1~15.9)	10.0 (7.7~12.3)	11.7 (7.0~17.7)	0.547
**Hemoglobin (g/L)**	102 (91.5~112.5)	105.5 (94.8~117.5)	96 (91~109)	0.473
Platelet (*10^9/L)	168 (90~232)	193.5 (79.8~283.5)	147 (97~198)	0.233
**NLR (%)**	18.5 (9.4~31.7)	23.8 (8.6~44.2)	16.6 (10.3~29.5)	0.459
**C-reactive protein (mg/L)**	109.1 (37.5~123.5)	119.1 (92.2~156.0)	210.9 (120.0~281.6)	**0.025**
**Procalcitonin (ng/mL)**	5.3 (0.3~19.3)	40.8 (3.69~100.0)	10.8 (5.3~53.9)	0.913
**PH**	7.48 (7.44~7.52)	7.48 (7.47~7.52)	7.5 (7.43~7.54)	0.742
**PaCO_2_ (mmHg)**	42.0 (36.0~70.4)	45.3 (38.0~78.0)	37.0 (32.0~80.0)	0.38
**HCO_3_^-^ (mmol/L)**	25.4 (20.7~29.7)	27.9 (22.8~31.9)	21.1 (15.4~26.5)	0.33
**PaO_2_/FiO_2_ (mmHg)**	228.6 (168.5~337.0)	229.5 (177.8~344.9)	228.6 (143~266.7)	0.482
**Lactate (mmol/L)**	1.6 (1.25~2.85)	2.1 (1.4~3.2)	5.2 (2.1~6.0)	**0.048**
**SOFA score**	9.0 (5.5~12.0)	7.5 (4~11.3)	11.0 (9-12)	**0.015**
**APACHE II score**	14.0 (10.5~28.5)	28.4 (13.0~29.5)	32.0 (25.0~33.8)	0.118
**Total cholesterol (mmol/L)**	3.0 (1.8~4.1)	3.2 (2.1~4.1)	2.1 (1.3~4.2)	0.375
**HDL-C (mmol/L)**	0.9 (0.6~1.2)	1.0 (0.8~1.3)	0.8 (0.3~1.0)	0.067
**LDL-C (mmol/L)**	1.6 (0.8~2.4)	1.6 (1.0~2.9)	0.9 (0.8~2)	0.415
**TBIL (µmol/L)**	14.8 (9.5~53.2)	85.6 (11.3~181.7)	32.1 (15.5~64.2)	0.511
**Creatinine (µmol/L)**	111.0 (51.5~186.9)	146.7 (84.9~213.1)	295.0 (142.9~398.2)	0.189
**Targeted metabolic analysis**				
**POVPC-D1 (ng/µL)**	292.0 (254.7~456.0)	270.0 (239.1~322.0)	451.0 (289.8~635.1)	**0.002**
**POVPC-D3 (ng/µL)**	313.7 (232.6~505.7)	239.5 (207.2~369.3)	406.4 (313.7~636.0)	**0.003**
**POVPC-D7 (ng/µL)^▲^**	288.7 (209.5~458.7)	296.4 (225.5~435.6)	440.0 (288.7~584.9)	0.205
**PGPC-D1, detected, n (%)**	5 (20.0)	1 (7.1)	4 (36.4)	0.133
**PGPC-D3, detected, n (%)**	6 (24.0)	4 (28.6)	2 (18.2)	0.661
**PGPC-D7, detected, n (%)^▲^**	4 (16.0)	2 (14.3)	2 (28.6)	0.574

NE, neutrophil granulocyte; NLR, neutrophil to lymphocyte ratio; PaCO_2_, arterial carbon dioxide tension; PaO_2_, arterial oxygen tension; FiO_2_, fraction of inspired oxygen; SOFA, sequential organ failure assessment; APACHE II, Acute Physiology and Chronic Health Evaluation II; HDL-C, high-density lipoprotein cholesterol; LDL-C, low-density lipoprotein cholesterol; TBIL, total bilirubin; POVPC-D1/D3/D7, the levels of 1-palmitoyl-2- (5-oxovaleroyl)-sn-glycero-phosphatidylcholine in HDL at Day 1 or Day 3 or Day 7; PGPC-D1/D3/D7: the detected number of subjects of 1-palmitoyl-2-glutaroyl-sn-glycero-phosphatidylcholine at Day 1 or Day 3 or Day 7; ▲, a total of 21 plasma samples (14 survivors and 7 non-survivors) were collected due to the death of 4 patients at Day 7. p value <0.05 was in bold, underline text denotes statistically significant index.

**Table 3:** Complications and treatments of septic survivors and non-survivors.

Variables	Total (n=25)	Sepsis (n=25)		
		Survivors (n=14)	Non-survivors (n=11)	P value
**Complications, n (%)**				
**Acute kidney injury**	6 (24.0)	2 (14.29)	4 (36.36)	0.199
**Liver dysfunction**	7 (28.0)	4 (28.57)	3 (27.27)	1
**Acute heart failure**	3 (12.0)	1 (7.14)	2 (18.18)	0.565
**Gastrointestinal bleeding**	7 (28.0)	3 (21.43)	4 (36.36)	0.656
**Pleural effusion**	10 (40.0)	7 (50)	3 (27.27)	0.414
**Treatment and progress, n (%)**				
**IMV**	14 (56.0)	7 (50)	7 (63.64)	0.589
**ECMO**	1 (4.0)	1 (7.14)	0	0.366
**CRRT**	4 (16.0)	1 (7.14)	3 (27.27)	0.288
**Vasopressor**	16 (64.0)	8 (57.14)	8 (72.73)	0.677
**Sedative**	18 (72.0)	11 (78.57)	7 (63.64)	0.656
**Parenteral nutrition**	12 (48.0)	5 (35.71)	7 (63.64)	0.238
**ICU days**	12.0 (8.0~17.0)	11.0 (8.0~19.3)	12.0 (7.0~17.0)	0.68
**Ventilator-free days**	24.0 (17.0~26.0)	24.5 (20.3~26.0)	23.0 (15.0~26.0)	0.678

IMV, invasive mechanical ventilation; ECMO, extracorporeal membrane oxygenation; CRRT, continuous renal replacement therapy; ICU, intensive care unit.

### The Correlation of HDL POVPC Level With SOFA Score and Serum CRP and Lactate Levels

**Figure 4:**
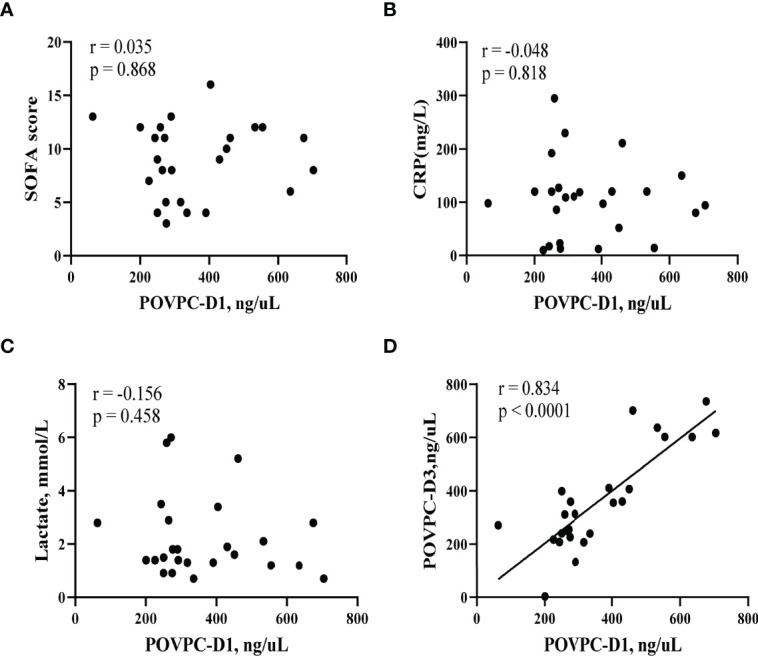
Correlation of HDL POVPC level with SOFA score and CRP and lactate levels. Scatter diagram for **(A)** correlation analysis between HDL POVPC-D1 and SOFA; **(B)** correlation analysis between HDL POVPC-D1 and CRP; **(C)** correlation analysis between HDL POVPC-D1 and lactate; **(D)** correlation analysis between HDL POVPC-D1 and HDL POVPC-D3. POVPC, 1-palmitoyl-2-(5-oxovaleroyl)-sn-glycero-phosphatidylcholine; POVPC-D1, POVPC at Day 1; POVPC-D3, POVPC at Day 3; HDL, high-density lipoprotein; SOFA, sequential organ failure assessment; CRP, C reactive protein.

To examine if the POVPC level would be correlated with the clinical conditions at the septic onset, we investigated the correlations of POVPC level with SOFA score, CRP, and lactate level. There were no statistical correlations between POVPC-D1 and these indexes (SOFA: r = 0.035, p = 0.868; CRP: r = -0.048, p = 0.818; lactate: r = -0.156, p = 0.458, respectively) (**Figures 4A–C**). Notably, there was a significant positive correlation between POVPC-D1 and POVPC-D3, suggesting an unreversible remodeling of HDL lipids (r = 0.834, p < 0.0001) (**Figure 4D**).

### The Correlation of HDL-POVPC Level With Plasma Cytokines During the Course of Sepsis

To examine the correlation between HDL-POVPC level and inflammatory conditions during sepsis, we measured the levels of 38 plasma cytokines by Luminex (**Supplementary Tables S4–S6**) and further evaluated the correlation of HDL-POVPC level with these cytokines at Day 1 and Day 3 after septic onset (**Table 4**). The correlation assay only showed that the IL-15 level was positively correlated with the level of POVPC at Day 1 (r = 0.44, p = 0.03). At Day 3, IL-15 and IL-18 levels were positively correlated with HDL-POVPC level (IL-15: r = 0.42, p = 0.04; IL-18: r = 0.53, p = 0.01), while the macrophage-derived chemokine (MDC) level was negatively correlated with HDL-POVPC level (r = -0.62, p = 0.001) (**Table 4**). In addition, compared with healthy controls, septic patients showed a significant change of the plasma cytokines profile at Day 1 and Day 3, in which the levels of IL-15, IL-18, and MDC were markedly altered at both Day 1 and Day 3 (**Supplementary Tables S4, S5**). Furthermore, compared to survivors, non-survivors exhibited increased levels in IL-18, MCP-1, M-CSF, PDGF-AA, and PDGF-AB/BB; the IL-6, IL-8, and M-CSF were increased only at Day 3 and RANTES was decreased only at Day 1 (**Supplementary Table S6**). These results suggested that HDL-POVPC level was not predominantly correlated with inflammatory conditions during the course of sepsis.

**Table 4:** Correlation analysis between the level of POVPC in HDL and cytokines.

Cytokines-D1/D3	POVPC-D1		POVPC-D3	
	r	p-value	r	p-value
**sCD40L**	-0.10	0.65	-0.18	0.40
**G-CSF**	-0.23	0.27	-0.07	0.72
**GRO-a**	-0.15	0.48	0.02	0.91
**IFN-a**	0.09	0.68	0.00	1.00
**IFN-r**	-0.02	0.94	0.04	0.84
**IL-1a**	0.13	0.52	0.13	0.54
**IL-1beta**	-0.01	0.96	0.02	0.94
**IL-1RA**	-0.11	0.60	0.29	0.15
**IL-2**	0.11	0.61	0.11	0.61
**IL-4**	0.24	0.25	0.39	0.06
**IL-5**	0.09	0.67	0.10	0.62
**IL-6**	-0.19	0.35	-0.02	0.92
**IL-7**	0.00	1.00	-0.02	0.91
**IL-8**	-0.02	0.91	0.18	0.40
**IL-9**	0.06	0.79	0.03	0.90
**IL-10**	0.25	0.22	0.21	0.32
**IL-13**	0.10	0.63	-0.01	0.97
**IL-15**	**0.44**	**0.03**	**0.42**	**0.04**
**IL-17A**	0.35	0.08	0.27	0.18
**IL-17E**	-0.02	0.94	0.18	0.40
**IL-17F**	0.31	0.14	0.19	0.36
**IL-18**	0.29	0.16	**0.53**	**0.01**
**IL-22**	-0.14	0.50	0.03	0.87
**IL-27**	-0.39	0.06	0.02	0.93
**IP-10**	-0.02	0.92	0.04	0.86
**MCP-1**	-0.12	0.56	-0.01	0.96
**MCP-3**	-0.04	0.84	-0.07	0.73
**M-CSF**	0.08	0.69	0.23	0.26
**MDC**	-0.30	0.15	**-0.62**	**0.001**
**MIG**	-0.06	0.76	0.06	0.77
**MIP-1a**	-0.03	0.88	-0.02	0.93
**MIP-1b**	-0.10	0.62	-0.28	0.18
**PDGFAA**	-0.31	0.13	-0.22	0.29
**PDGFBB**	-0.27	0.19	-0.24	0.24
**RANTES**	-0.31	0.13	-0.15	0.48
**TGF-a**	0.29	0.16	0.23	0.27
**TNF-a**	0.03	0.87	0.37	0.07
**TNF-b**	0.22	0.29	0.04	0.86

Cytokines-D1/D3, the levels of cytokines in plasma of patients on Day 1 or Day 3 of sepsis. POVPC-D1/D3, the levels of 1-palmitoyl-2-(5-oxovaleroyl)-sn-glycero-phosphatidylcholine on Day 1 or Day 3 of sepsis. p value <0.05 was in bold, underline text denotes statistically significant index.

### Predictive Value of HDL POVPC for 28-Day Mortality of Septic Patients

**Figure 5:**
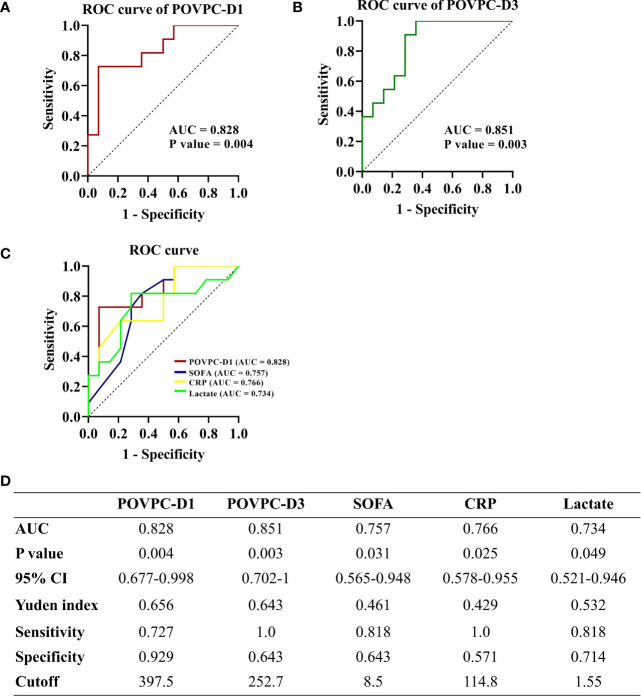
Predictive ability of HDL POVPC for 28-day mortality of septic patients. ROC curve for HDL POVPC-D1 and POVPC-D3 in diagnosis of the sepsis **(A, B)**. ROC curves for POVPC-D1, SOFA score, CRP, and lactate in prediction of the 28-day mortality in septic patients **(C)**, and the Youden’s index in order to maximize both sensitivity and specificity **(D)**. ROC, receiver operating characteristic; AUC, area under the curve; CI, confidence interval; HDL, high-density lipoprotein; SOFA, sequential organ failure assessment; CRP, C-reaction protein; POVPC, 1-palmitoyl-2-(5-oxovaleroyl)-sn-glycero-phosphatidylcholine; D1, Day 1; D3, Day 3.

The ROC curves for HDL POVPC-D1 and POVPC-D3, SOFA score, plasma CRP, and lactate levels were plotted to compare the accuracy and specificity for the prediction of 28-day mortality. The area under the curve (AUC) for POVPC-D1 was 0.828 (p = 0.004, 95% CI: 0.677-0.998) (**Figure 5A**), for POVPC-D3 was 0.851 (p = 0.003, 95% CI: 0.702-1) (**Figure 5B**), for SOFA score was 0.757 (p = 0.031, 95% CI: 0.565-0.948), for CRP was 0.766 (p = 0.025, 95% CI: 0.578-0.955), and for lactate was 0.734 (p = 0.049, 95% CI: 0.521-0.946) (**Figure 5C**), suggesting the better predictive values of POVPC-D1/3 vs. other indexes (**Figure 5**).

We further selected cutoff values based on the highest Youden’s index in order to maximize both sensitivity and specificity (Youden’s index=sensitivity+specificity-1). A POVPC-D1 cutoff value of 397.5 ng/μL had a sensitivity of 72.7% and a specificity of 92.9%. A POVPC-D3 cutoff value of 252.7 ng/μL had a sensitivity of 100.0% and a specificity of 64.3%. Septic patients were regrouped by these cutoff values to validate their prediction efficacy for 28-day mortality, as well as the severity at sepsis onset, including SOFA score, APACHE II score, and serum CRP and lactate levels. The POVPC-D1 and POVPC-D3 displayed a predictive efficacy for 28-day mortality, but not the sepsis onset severity (POVPC-D1: p = 0.002; POVPC-D3, p = 0.001) (**Table 5**), suggesting unique clinical merits of HDL POVPC as a biomarker for prognosis.

**Table 5:** Hospital outcomes by HDL POVPC-D1 and HDL POVPC-D3 cutoff values in septic patients.

Outcomes	POVPC levels in HDL at Day 1 after septic diagnosis		P value
	POVPC < 397.5 ng/µl	POVPC > 397.5 ng/µl	
	(n=16)	(n=9)	
**APACHE II score**	13.0 (8.5-25.8)	27.0 (12.5-32.5)	**0.041**
**28-day mortality, n (%)**	3 (18.8)	8 (88.9)	**0.002**
**SOFA score**	7 (4.5-10)	11 (8-12)	0.087
**C-reactive protein**	86.0 (14.8-120)	114.0 (83.7-114.3)	0.979
**HDL-C**	0.9 (0.8-1.2)	0.9 (0.3-1.4)	0.368
**Lactate**	1.45 (1.3-2.9)	1.9 (1.2-3.1)	0.821
**Outcomes**	**POVPC levels in HDL at Day 3 after septic diagnosis**		**P value**
	**POVPC < 252.7 ng/µL**	**POVPC > 252.7 ng/µL**	
	**(n=9)**	**(n=16)**	
**APACHE II score**	14.0 (10.0-28.5)	18.5 (10.3-29.3)	0.715
**28-day mortality, n (%)**	0 (0)	11 (68.8)	**0.001**
**SOFA score**	8 (5-10)	11 (6.5-12)	0.17
**C-reactive protein**	109.3 (20.3-119.4)	109.0 (59.1-144.3)	0.383
**HDL-C**	0.9 (0.8-1.2)	0.9 (0.4-1.3)	0.914
**Lactate**	1.4 (11-2.2)	1.9 (1.2-3.3)	0.245

APACHE II, Acute Physiology and Chronic Health Evaluation II; SOFA, sequential organ failure assessment; HDL-C, high-density lipoprotein cholesterol. p value < 0.05 was in bold.

### Enter Logistic Regression Analysis to Examine the Independent Correlations

Enter logistic regression was performed to identify the risk factors for the 28-day mortality, factors with p<0.05 in univariate logistic regression were in turn subjected to multivariate logistic regression. The univariate logistic regression analysis showed POVPC-D1, POVPC-D3, SOFA, and CRP were significantly associated with 28-day mortality of septic patients (OR: 1.011, 95% CI: 1.002-1.02, p = 0.017; OR: 1.009, 95% CI: 1.001-1.016, p = 0.018; OR: 1.418, 95% CI: 1.035-1.943, p = 0.03; OR: 1.018, 95% CI: 1.001-1.036, p = 0.04). We constructed two multivariate binary regression models, Model I included CRP, SOFA score, and POVPC-D1, Model II included CRP, SOFA score, and POVPC-D3. The goodness-of-fit for these two regression models was evaluated with the Hosmer Lemeshow test. For Model I, the p value of the Hosmer Lemeshow goodness-of-fit test was 0.959; for Model II, the p value was 0.648; both p values were greater than 0.05, which indicated these two models were a good fit. The -2log likelihood value of Model I was 10.06, and the Nagelkerke R Square of Model I was 0.83; for Model II they were 13.38 and 0.76, respectively. These results suggested that our fitted logistic regression models were reliable. Multivariate logistic regression results confirmed both POVPC-D1 and POVPC-D3 were significantly associated with 28-day mortality of septic patients (POVPC-D1: OR=1.018, 95% CI: 1.0-1.036, p = 0.046; POVPC-D3: OR=1.012, 95% CI: 1.001-1.023, p = 0.035) (**Table 6**).

**Table 6:** Univariate and multivariate logistic regression analysis of independent risk factors for 28-day mortality in septic patients.

Variables	Univariate analysis		P value	Multivariate analysis		P value
	OR	95% CI		OR	95% CI	
**C-reactive protein**	1.018	1.001-1.036	**0.04**	1.036	0.994-1.08	0.091
**SOFA score**	1.418	1.035-1.943	**0.03**	1.664	0.746-3.71	0.213
**POVPC-D1**	1.011	1.002-1.02	**0.017**	1.018	1.0 -1.036	**0.046**
**Lactate**	2.104	0.966-4.582	0.061			
**Variables**	**Univariate analysis**		**P value**	**Multivariate analysis**		**P value**
	**OR**	**95% CI**		**OR**	**95% CI**	
**C-reactive protein**	1.018	1.001-1.036	**0.04**	1.029	0.995-1.063	0.094
**SOFA score**	1.418	1.035-1.943	**0.03**	1.453	0.895-2.36	0.131
**POVPC-D3**	1.009	1.001-1.016	**0.018**	1.012	1.001-1.023	**0.035**
**Lactate**	2.104	0.966-4.582	0.061			

SOFA, sequential organ failure assessment; OR, odds ratio; CI, confidence interval; POVPC-D1, 1-palmitoyl-2-(5-oxovaleroyl)-sn-glycero-phosphatidylcholine at Day 1; POVPC-D3, 1-palmitoyl-2-(5-oxovaleroyl)-sn-glycero-phosphatidylcholine at Day 3. p value <0.05 was in bold.

## Discussion

Our study, for the first time, confirmed the marked increase of POVPC level in the HDL particle during the course of sepsis. We further indicated that the HDL-POVPC levels at Day 1 and Day 3 after septic onset were independent risk factors for poor prognosis of septic patients. POVPC-D1: 397.5 ng/μL and POVPC-3: 252.7 ng/μL were significantly correlated with the 28-day mortality of septic patients.

The Ox-PAPCs, including POVPC and PGPC have been involved in the chronic atherosclerotic pathogenesis, for decades, by modulating inflammatory response, endothelial barrier function, thrombosis, and angiogenesis (22, 23). Recently, POVPC was detected in the plasma of septic and ALI patients and the increase in POVPC generation was observed in severe ALI with marked vascular leakage and enhanced inflammation (24–26). In this study, we indicated a sustained increase of POVPC in the HDL particle from septic non-survivors at Day 1 and 3 and the HDL-POVPC levels were positively correlated with some key pro-inflammatory cytokines. The HDL-POVPC level at Day 7 failed to achieve significance, which might be due to the complicated conditions after several days of treatments intervene. Given the importance of HDL in anti-inflammation, these observations for the first time suggested that the Ox-PAPCs in HDL, other than in LDL, likely contribute to deregulated inflammation in sepsis. Moreover, our results exhibited a persistent increase of HDL POVPC from Day 1 to Day 7 during the course of sepsis, implying an unreversible remodeling of HDL lipids. Interestingly, we also detected the POVPC in plasma HDL from healthy controls (**Table 1**). A previous study showed an age-dependent increase of POVPC production, suggesting an association between oxidative HDL remodeling and aging (26). In our study, the constitute of HDL-POVPC in healthy controls is likely due to their elder ages (71 [59~82.5] years old).

Previous studies have demonstrated the contribution of Ox-PAPC to the deleterious function of oxidized LDL in atherosclerosis and the oxidized LDL has been found to be increased in patients with severe sepsis (27, 28). Of note, the dysfunction of HDL was associated with the increase of oxidized LDL, owing to its essential role in preventing the formation of the LDL-derived oxidized phospholipids as well as inactivating formed oxidized phospholipids by enzymatic and non-enzymatic mechanisms (29, 30). The oxidation degree of HDL itself would be more sensitive than that of LDL in response to septic disorder. Consistently, our results showed the persistent increase of HDL-POVPC in septic patients at Day 1 and 3, which was significantly correlated with pro-inflammatory cytokines and exhibited better predictive abilities for 28-day mortality of septic patients than SOFA, CRP, and lactate. These observations suggested the significant role of HDL-POVPC in sepsis. Thus, we hypothesize that the higher levels of oxidized LDL could be associated with the enhanced HDL-oxidation. These pilot results also imply that HDL-POVPC could be a potential biomarker of sepsis outcome.

The SOFA score, plasma CRP, and lactate levels normally present during the clinical conditions at septic onset include inflammatory stress and organ injuries. Of note, our correlation analyses failed to show significant correlation between HDL-POVPC levels and these indexes, although they were associated with poor prognosis of septic patients. This might be due to that HDL-POVPC represents the dysfunction of HDL including anti-inflammatory and endothelial protection. Our further correlation analysis showed HDL-POVPC levels were significantly correlated with several critical inflammatory cytokines including IL-15, IL-18, and MDC which were dramatically changed in septic patients, although HDL-POVPC was not predominantly correlated with inflammatory conditions. Notably, IL-18 level was dramatically increased in septic non-survivors vs. survivors at both Day 1 and Day 3. This is consistent with previous findings that the increase of plasma IL-18 level has been shown as a biomarker for adverse outcomes of sepsis (31, 32). The positive correlation of HDL-POVPC levels with IL-18 endorsed the prognostic value of HDL-POVPC for sepsis. In addition, MDC, as a CC chemokine involved in dendritic cell and lymphocyte homing, can ameliorate systemic tissue inflammation. The decrease of MDC level is associated with poor outcome of sepsis (33). Herein, our results showed HDL-POVPC was negatively associated with MDC that was significantly decreased in septic patients. These observations suggested the unique contribution of HDL-POVPC in inflammatory disorder, which might be associated with poor prognosis of sepsis.

Sepsis is involved with complex interactions between microbes, immune cells, and endothelium. It has been shown the truncated Ox-PAPCs, including POVPC and PGPC, can induce vascular leaking and exacerbate inflammation, due to their direct pro-inflammatory effects on both endothelial cell and macrophages (24, 34, 35). The deleterious effects of POVPC on endothelial function have been highlighted for decades in cardiovascular diseases (36–38). Therefore, the variety of POVPC function could be an explanation for why HDL POVPC was not significantly correlated with these SOFA scores, plasma CRP, and other pro-inflammatory cytokines enhanced in septic patients. Our finding further suggested the deleterious effects of HDL-POVPC on endothelial barrier should be considered in clinical therapeutic strategy for septic patients, such as respiratory disorders and vascular leaking.

It has been shown that a variety of HDL protective functions (anti-oxidative, anti-inflammatory, and vasoprotective effects) were impaired upon the pathological processes with oxidative stress, especially in acute sepsis (7, 39). The oxidative modification of HDL apoproteins, such as the apoprotein A-I, has been proposed as a key molecular mechanism leading to HDL dysfunction (40). However, recent evidence indicates oxidized phospholipids (Ox-PLs) in HDL can modify apoproteins *via* direct cross-linking leading to their dysfunction (41). Oxidized phospholipids could be more sensitive than apoprotein modification to represent HDL dysfunction. Consistently, in our study, the HDL-POVPC seemed to be more efficient to predict 28-day mortality than SOFA score and plasma CRP and lactate levels indicated by ROC curve (AUC: POVPC-D1, 0.828 and POVPC-D3, 0.851 vs. SOFA, 0.757, CRP 0.766, and lactate, 0.734). Furthermore, we evaluated the cutoff point of POVPC levels for prognostic prediction in septic patients: POVPC-D1: 397.5 ng/μL with a sensitivity of 72.7% and a specificity of 92.9% and POVPC-D3: 252.7 ng/μL with a sensitivity of 100.0% and a specificity of 64.3%. The validation cohort with larger sample sizes is worthy of a study to evaluate the predictive merits of POVPC for sepsis.

The decrease of HDL-C level has become a poor prognostic factor for sepsis, owing to its dramatic decrease in these patients (42–44). Our septic cohort also showed significantly decreased levels of HDL-C at admission vs. healthy controls (0.9 vs. 1.5 mmol/L, p = 0.001), but there was no significant difference between survivors and non-survivors. This inconsistency might be due to the limited sample size, as indicated by the obvious group variation (survivors: 1.0 (0.8~1.3); non-survivors: 0.8 (0.3~1.0) mmol/L). In contrast, this cohort exhibited significant difference in HDL POVPC levels between survivors and non-survivors, suggesting that the HDL quality remodeling seemed to be more sensitive than quantity change in the course of sepsis.

## Conclusion

Our study, for the first time, showed a marked increase of POVPC level in the HDL particle from septic patients and such increase is persistent during the course of sepsis. We further indicated that the POVPC levels at Day 1 and Day 3 after sepsis onset were independent risk factors for poor prognosis of septic patients. POVPC-D1: 397.5 ng/μL and POVPC-3: 252.7 ng/μL were significantly correlated with the 28-day mortality of septic patients. Our finding further endorses the necessity to explore the quality remodeling of HDL in response to septic stress. Future studies embracing larger sample sizes is required to provide solid evidence for the predictive merits of POVPC for sepsis.

## Limitations

Several limitations should be considered in our present research. First, our study was carried out at a single center, results cannot be extrapolated. Second, our sample size was relatively small, which may have affected our results. The outcome in regression models for the prognostic value of POVPC levels was rather low, which limited generalizability of our results. Third, our recruited septic patients were predominantly infected with bacteria, thus our results may not be suitable for viral infected septic patients.

## Data Availability Statement

The original contributions presented in the study are included in the article/**Supplementary Material**. Further inquiries can be directed to the corresponding authors.

## Ethics Statement

The studies involving human participants were reviewed and approved by The Ethics Committee of Beijing ChaoYang Hospital (2021-ke-313). The patients/participants provided their written informed consent to participate in this study.

## Author Contributions

ZHL: Formal analysis, writing-original draft. XS and ZTL: Data processing. BP Writing-review and editing. YM: Conceptualization and design. JJ: Writing-review and editing. All authors contributed to the article and approved the submitted version.

## Funding

This work was supported by Reform and Development Program of Beijing Institute of Respiratory Medicine (2021).

## Conflict of Interest

The authors declare that the research was conducted in the absence of any commercial or financial relationships that could be construed as a potential conflict of interest.

## Publisher’s Note

All claims expressed in this article are solely those of the authors and do not necessarily represent those of their affiliated organizations, or those of the publisher, the editors and the reviewers. Any product that may be evaluated in this article, or claim that may be made by its manufacturer, is not guaranteed or endorsed by the publisher.

## Abbreviations

HDL (-C), high-density lipoprotein (-cholesterol); LDL (-C), low-density lipoprotein (-cholesterol); VLDL (-C), very low-density lipoprotein (-cholesterol); CHOL, cholesterol; Ox-PLs, oxidized phospholipids; UHPLC-MS/MS, ultra-high performance liquid chromatography coupled to tandem mass spectrometry system; PAPC, 1-palmitoyl-2-arachidonoyl-sn-glycero-3-phosphatidylcholine; Ox-PAPCs, oxidized PAPCs; POVPC, 1-palmitoyl-2-(5-oxovaleroyl)-sn-glycero-phosphatidylcholine; PGPC, 1-palmitoyl-2-glutaroyl-sn-glycero-phosphatidylcholine; D1, at Day 1; D3, at Day 3; D7, at Day 7; Ox-LDL, oxidized low-density lipoprotein; apoA-1, apoprotein A-1; apoB100, apoprotein B 100; BMI, body mass index; PCT, procalcitonin; CRP, C-reactive protein; SOFA score, Sequential organ failure assessment score; APACHE II score, acute physiology and chronic health evaluation II score; CRRT, continuous renal replacement therapy; IQRs, interquartile ranges; ROC, receiver operating characteristic; AUC, area under the curve; ALI, acute lung injury.
