# Supplementary material for: The Levels of Oxidized Phospholipids in High-Density Lipoprotein During the Course of Sepsis and Their Prognostic Value
Source: Front Immunol. 2022 May 3;13:893929. doi: 10.3389/fimmu.2022.893929 (PMC9111014; doi:10.3389/fimmu.2022.893929)
Supplement: Supplementary file 1 [file DataSheet_1.pdf]

Supplementary figure 1. The purity of HDL fractions isolated from plasma.

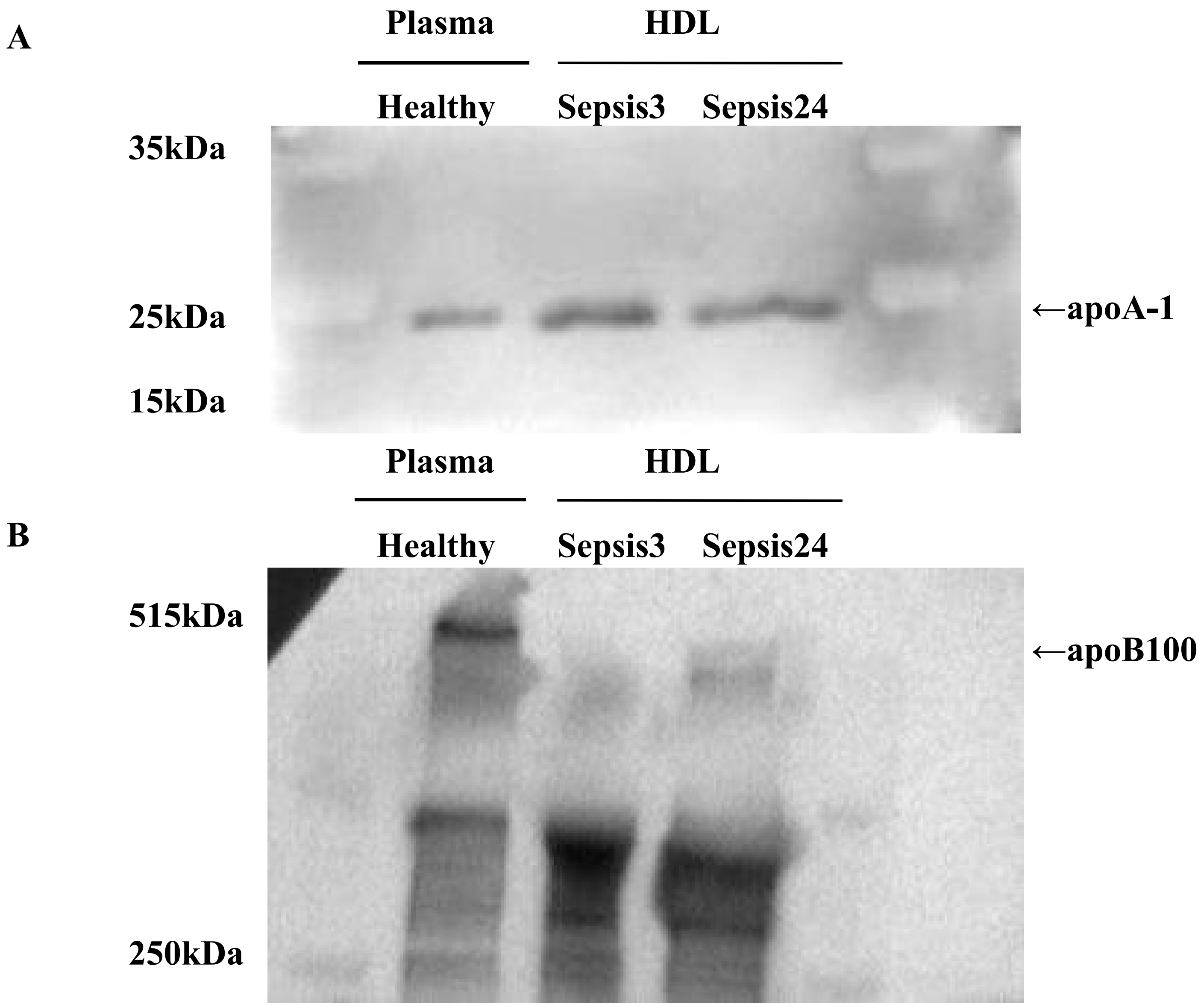

Supplementary figure 1. The purity of HDL fractions isolated from plasma. Measurements of apoA-1 (A) and apoB100 (B) by immunoblot analysis; line 1, plasma from healthy subject; line 2 and 3, HDL extracted from two septic patients. The most abundant protein component of high density lipoprotein, apoA-1 ,were detected in both plasma and extracted HDL samples (25kDa); apoB100, which presents predominantly in low density lipoprotein, was only detected in the plasma of healthy subject (515kDa).

Supplementary figure 2. The lipid profiles of POVPC and PGPC in HPLC

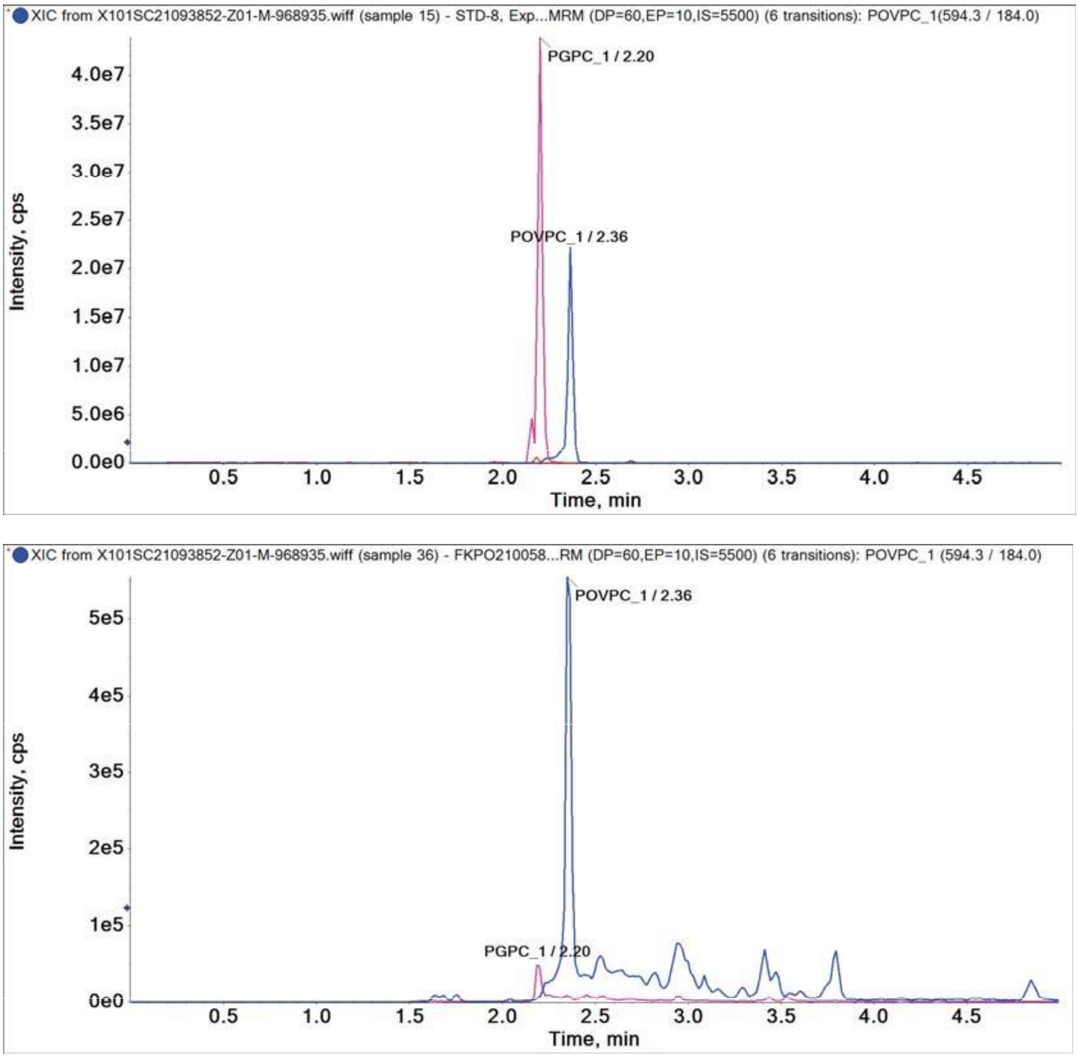

Supplementary figure 2. The lipid profiles of POVPC and PGPC in HPLC. POVPC and PGPC of standard substances (top) and the HDL samples of enrolled subjects (bottom) as obtained by ultra-high performance liquid chromatography coupled to tandem mass spectrometry (UHPLC-MS/MS) system after dephosphorylation of the lipid extracts.

Supplementary figure.3. The standard curve of POVPC (up) and PGPC (bottom).

Regression Equation: y = 0.03769 x + 0.00223 (r = 0.99641, r² = 0.99283) (weighting: 1 / x^2)

Expected Concentration	Number of Values	Mean Calculated Concentration (No data for Analyte Unit)	% Accuracy	Std. Deviation	%CV
0.200	1 of 1	0.203	101.3	N/A	N/A
1.000	1 of 1	0.966	96.6	N/A	N/A
2.000	1 of 1	1.919	95.9	N/A	N/A
10.000	1 of 1	9.360	93.6	N/A	N/A
20.000	1 of 1	17.628	88.1	N/A	N/A
100.000	1 of 1	112.164	112.2	N/A	N/A
200.000	1 of 1	218.668	109.3	N/A	N/A
1000.000	1 of 1	1029.630	103.0	N/A	N/A

Analyte Name: **1-palmitoyl-2-(5-oxo-valeroyl)-sn-glycero-3-phosphocholine**

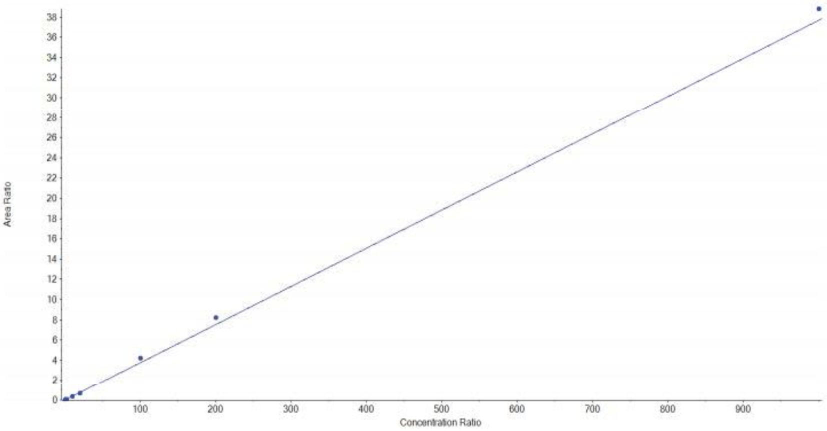

Regression Equation: y = 0.07180 x + 0.00387 (r = 0.99710, r² = 0.99421) (weighting: 1 / x^2)

Expected Concentration	Number of Values	Mean Calculated Concentration (No data for Analyte Unit)	% Accuracy	Std. Deviation	%CV
0.200	1 of 1	0.201	100.4	N/A	N/A
1.000	1 of 1	1.012	101.2	N/A	N/A
2.000	1 of 1	1.897	94.9	N/A	N/A
10.000	1 of 1	9.672	96.7	N/A	N/A
20.000	1 of 1	17.586	87.9	N/A	N/A
100.000	1 of 1	106.469	106.5	N/A	N/A
200.000	1 of 1	223.721	111.9	N/A	N/A
1000.000	1 of 1	1005.305	100.5	N/A	N/A

Analyte Name: **1-palmitoyl-2-glutaryl-sn-glycero-3-phosphocholine**

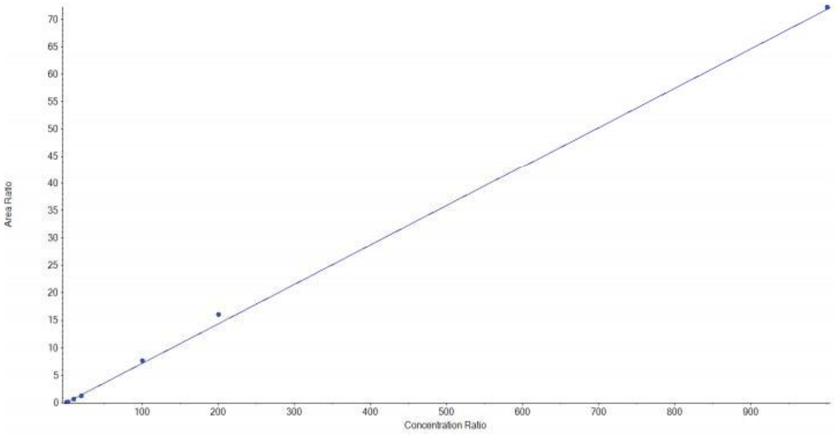

Supplemtary table 1. Ultra-performance liquid chromatography-mass spectrometry (UPLC-MS) parameters.

Component Name	Q1	Q3	RT(min)	DP	CE
**POVPC**	594.3	184.0	2.37	60	35
**PGPC**	610.3	184.1	2.27	60	35
**Decanoic acid-d19**	190.2	190.2	2.21	-40	-12

Abbreviations: Q1, parent ion; Q3, daughter ion; RT, retention time; DP, declustering potential; CE, collision energy; POVPC, 1-palmitoyl-2-(5-oxovaleroyl)-sn-glycerophosphatidylcholine; PGPC, 1-palmitoyl-2glutaroyl-sn-glycero-phosphatidylcholine.

Supplementary table 2. The raw data of POVPC and PGPC in septic patients at day 1 and day 3.

Sample	POVPC, ng/uL	PGPC, ng/uL
S3_D1	554.9438619	ND
S4_D1	390.7719568	ND
S6_D1	243.4011403	ND
S8_D1	250.5762702	37.05907616
S11_D1	291.9591502	ND
S14_D1	276.3755924	ND
S17_D1	63.01494226	ND
S18_D1	335.7371426	ND
S1_D1	704.5381686	6.08591643
S5_D1	635.1259231	2.853881866
S9_D1	317.4669445	ND
S10_D1	404.1871295	4.405904366
S16_D1	430.5331601	ND
S19_D1	258.9101024	ND
S20_D1	289.8005034	ND
S21_D1	676.69844	267.3932044
S22_D1	200.5884339	ND
S25_D1	226.1650019	ND
S26_D1	274.9944258	ND
S27_D1	250.3497739	ND
S29_D1	264.8695409	ND
S23_D1	532.9846892	ND
S24_D1	271.2909125	ND
S28_D1	451.0218125	ND
S30_D1	460.9623493	ND

Sample	POVPC, ng/uL	PGPC, ng/uL
S3_D3	601.4327327	ND
S4_D3	410.2016747	9.908563229
S6_D3	207.523006	ND
S8_D3	3398.762463	334.858432
S11_D3	132.0944676	ND
S14_D3	359.5238631	ND
S17_D3	270.9464005	ND
S18_D3	238.9443749	ND
S1_D3	616.2690508	33.47930282
S5_D3	601.2597605	ND
S9_D3	206.3039843	0.010718617
S10_D3	354.8683355	ND
S16_D3	359.8923762	ND
S19_D3	311.35897	ND
S20_D3	313.6740404	ND
S21_D3	1735.147132	40.3747748
S22_D3	2.975386819	ND
S25_D3	217.3198567	ND
S26_D3	226.2628772	6.405633971
S27_D3	240.1348889	ND
S29_D3	250.6055709	ND
S23_D3	636.0196279	ND
S24_D3	254.7667466	ND
S28_D3	406.3974211	ND
S30_D3	700.7194368	ND

Abbreviations: POVPC, 1-palmitoyl-2- (5-oxovaleroyl) -sn-glycero-phosphatidylcholine; PGPC, 1-palmitoyl-2-glutaroyl-sn-glycero-phosphatidylcholine; S, sepsis (the number after S is the number of enrolled subjects); D1, day 1; D3, day 3; ND, not detected.

Supplementary table 3. The raw data of POVPC and PGPC in septic patients at day 7 and in healthy controls.

Sample	POVPC, ng/uL	PGPC, ng/uL
S3_D7	500.4954996	ND
S4_D7	235.1403933	ND
S6_D7	284.7114605	ND
S8_D7	833.9197547	33.53729728
S11_D7	286.189145	ND
S14_D7	492.0307639	ND
S17_D7	227.8688609	ND
S18_D7	344.0326266	8.746077863
S5_D7	477.4306076	ND
S9_D7	200.1054838	ND
S10_D7	354.6448276	ND
S20_D7	440.0343002	ND
S21_D7	606.0294037	53.65683061
S22_D7	416.7603865	ND
S25_D7	218.0977924	ND
S26_D7	306.6801501	ND
S27_D7	218.3911802	ND
S29_D7	340.1656817	ND
S23_D7	200.8155417	ND
S24_D7	288.7384592	ND
S30_D7	584.930622	4.040431266

Sample	POVPC, ng/uL	PGPC, ng/uL
Hc_1	215.5714149	ND
Hc_2	233.4368046	ND
Hc_3	203.3519346	ND
Hc_4	235.3466416	ND
Hc_5	238.0638208	ND
Hc_6	276.1769488	ND
Hc_7	245.1474249	ND
Hc_8	211.5837358	ND
Hc_9	216.2728737	ND
Hc_10	206.3944797	ND

Abbreviations: POVPC, 1-palmitoyl-2- (5-oxovaleroyl) -sn-glycero-phosphatidylcholine; PGPC, 1-palmitoyl-2-glutaroyl-sn-glycero-phosphatidylcholine; S, sepsis (the number after S is the number of enrolled subjects) ; D7, day 7; Hc, healthy control (the number after Hc is the number of healthy controls) ; ND, not detected.

Supplementary table 4. Comparison of differences in cytokines between patients and healthy controls at day 1 of sepsis.

CytoKines pg/mL	Sepsis at day 1 (n=25)	Healthy controls (n=10)	P value
**sCD40L**	98.4 (35.1~158.3)	52.5 (36.7~58.5)	0.242
**G-CSF**	49.8 (22.9~373.5)	3.1 (1.3~9.1)	**0.001**
**GRO-α**	7.5 (3.5~12.3)	3.8 (2.6~6.8)	0.208
**IFN-α 2**	9.4 (6.3~21.8)	7.2 (5.8~12.9)	0.391
**IFN-ɤ**	1.0 (0.6~1.9)	0.6 (0.5~1.6)	0.214
**IL-1α**	3.5 (2.7~9.2)	2.7 (2.2~3.6)	0.104
**IL-1β**	6.3 (2.7~21.7)	2.9 (1.1~10.3)	0.177
**IL-1RA**	15.2 (8.3~62.6)	4.1 (3.0~5.0)	**<0.0001**
**IL-2**	0.3 (0.1~1.1)	0.2 (0.1~0.4)	0.476
**IL-4**	0.3 (0.1~0.7)	0.2 (0.1~0.4)	0.499
**IL-5**	2.2 (1.2~6.8)	2.7 (2.0~4.6)	0.841
**IL-6**	62.9 (16.1~98.5)	1.9 (1.1~3.4)	**<0.0001**
**IL-7**	0.4 (0.2~0.9)	0.3 (0.2~0.5)	0.401
**IL-8**	20.8 (6.6~41.1)	3.6 (2.9~4.5)	**0.002**
**IL-9**	0.6 (0.1~15.3)	0.2 (0.1~2.0)	0.228
**IL-10**	11.5 (1.0~59.3)	0.3 (0.2~0.4)	**<0.0001**
**IL-13**	5.7 (3.6~14.1)	4.8 (3.6~28.1)	1
**IL-15**	20.8 (10.9~39.5)	5.8 (5.4~6.6)	**<0.0001**
**IL-17A**	3.3 (1.6~9.3)	1.3 (1.1~1.7)	**0.013**
**IL-17E/IL-25**	322.6 (119.2~616.3)	292.5 (110.0~359.8)	0.361
**IL-17F**	17.3 (12.2~31.9)	12.6 (10.9~13.1)	0.053
**IL-18**	74.3 (38.6~138.4)	35.3 (25.2~47.2)	**0.003**
**IL-22**	2.9 (1.4~5.4)	2.5 (2.0~5.3)	0.884
**IL-27**	4813.0 (2027.3~6181.8)	1533.0 (804.2~1821.0)	**0.001**
**IP-10**	322.1 (141.3~599.0)	132.1 (107.4~169.2)	**0.02**
**MCP-1**	520.9 (306.8~769.2)	311.1 (294.0~355.5)	**0.028**
**MCP-3**	9.8 (6.9~16.2)	7.3 (6.4~19.7)	0.38
**M-CSF**	257.5 (70.3~608.9)	15.5 (12.4~23.8)	**<0.0001**
**MDC**	264.3 (150.0~416.7)	645.0 (598.1~788.8)	**<0.0001**
**MIG**	5381.5 (1908.8~10143.0)	1702.5 (1076.3~2318.3)	**0.009**
**MIP-1α**	17.3 (4.3~52.5)	6.2 (2.7~22.9)	0.165
**MIP-1β**	63.7 (44.3~94.7)	40.0 (30.7~52.2)	0.057
**PDGF-AA**	176.4 (80.8~458.2)	329.1 (225.2~506.4)	0.195
**PDGF-AB/BB**	2726.0 (956.8~6211.5)	5427.5 (4567.8~7056.3)	0.056
**RANTES**	2813.0 (1211.5~4436.0)	6211.0 (5387.5~7199.0)	**<0.0001**
**TGF-α**	4.5 (1.3~12.9)	1.4 (0.8~2.3)	**0.014**
**TNF-α**	48.6 (20.2~71.6)	14.3 (11.8~17.1)	**0.009**
**TNF-β**	1.3 (0.4~2.9)	2.1 (0.8~10.5)	0.361

Supplementary table 5. Comparison of differences in cytokines between patients and healthy controls at day 3 of sepsis.

Cytokines (pg/mL)	Sepsis at day 3 (n=25)	Healthy controls (n=10)	P value
**sCD40L**	103.2 (57.7~154.5)	52.5 (36.7~58.5)	**0.02**
**G-CSF**	28.6 (2.7~ 80.5)	3.1 (1.3~9.1)	0.051
**GRO-α**	4.5 (1.8~10.2)	3.8 (2.6~6.8)	0.942
**IFN-α2**	9.6 (7.2~20.4)	7.2 (5.8~12.9)	0.16
**IFN-ɤ**	1.0 (0.6~2.6)	0.6 (0.5~1.6)	0.188
**IL-1α**	3.9 (2.7~6.5)	2.7 (2.2~3.6)	0.112
**IL-1β**	6.7 (1.6~21.8)	2.9 (1.1~10.3)	0.432
**IL-1RA**	14.3 (8.0~27.1)	4.1 (3.0~5.0)	**<0.0001**
**IL-2**	0.3 (0.1~0.5)	0.2 (0.1~0.4)	0.371
**IL-4**	0.2 (0.2~0.8)	0.2 (0.1~0.4)	0.281
**IL-5**	6.9 (2.4~15.1)	2.7 (2.0~4.6)	0.1
**IL-6**	27.8 (10.4~86.6)	1.9 (1.1~3.4)	**<0.0001**
**IL-7**	0.3 (0.2~0.8)	0.3 (0.2~0.5)	0.812
**IL-8**	15.4 (4.6~33.7)	3.6 (2.9~4.5)	**0.003**
**IL-9**	0.6 (0.1~12.5)	0.2 (0.1~2.0)	0.228
**IL-10**	10.4 (2.2~35.5)	0.3 (0.2~0.4)	**<0.0001**
**IL-13**	6.4 (4.3~15.7)	4.8 (3.6~28.1)	0.547
**IL-15**	22.8 (9.7~34.7)	5.8 (5.4~6.6)	**<0.0001**
**IL-17A**	3.8 (1.4~13.4)	1.3 (1.1~1.7)	**0.03**
**IL-17E/IL25**	271.8 (106.8~607.0)	292.5 (110.0~359.8)	0.622
**IL-17F**	17.0 (11.2~29.9)	12.6 (10.9~13.1)	0.125
**IL-18**	74.6 (38.6~151.9)	35.3 (25.2~47.2)	**0.004**
**IL-22**	2.6 (1.4~5.1)	2.5 (2.0~5.3)	0.571
**IL-27**	3750.5 (1442.5~5661.3)	1533.0 (804.2~1821.0)	**0.005**
**IP-10**	248.9 (173.4~553.8)	132.1 (107.4~169.2)	**0.002**
**MCP-1**	406.4 (305.3~866.8)	311.1 (294.0~355.5)	0.112
**MCP-3**	10.3 (6.9~15.3)	7.3 (6.4~19.7)	0.476
**M-CSF**	171.0 (82.1~464.5)	15.5 (12.4~23.8)	**<0.0001**
**MDC**	218.8 (139.1~326.6)	645.0 (598.1~788.8)	**<0.0001**
**MIG**	4602.0 (2201.0~8944.0)	1702.5 (1076.3~2318.3)	**0.006**
**MIP-1α**	21.2 (6.5~3.2)	6.2 (2.7~22.9)	0.129
**MIP-1β**	66.6 (42.0~90.7)	40.0 (30.7~52.2)	**0.049**
**PDGF-AA**	306.4 (97.6~567.1)	329.1 (225.2~506.4)	0.648
**PDGF-AB/BB**	4355.0 (1366.0~9437.0)	5427.5 (4567.8~7056.3)	0.734
**RANTES**	2719.5 (853.8~4379.5)	6211.0 (5387.5~7199.0)	**<0.0001**
**TGF-α**	4.0 (1.6~10.0)	1.4 (0.8~2.3)	**0.018**
**TNF-α**	34.1 (19.7~56.0)	14.3 (11.8~17.1)	**0.002**
**TNF-β**	1.3 (0.6~4.5)	2.1 (0.8~10.5)	0.342

Supplementary table 6. Comparison of differences in cytokines between septic survivors and non-survivors at day 1

Cytokines pg/mL	Septic patients (n=25) at day 1		P value
	Survivors (n=14)	Non-survivors (n=11)	
**IL-6**	38.6 (3.5-86.4)	93.0 (30-311.1)	0.095
**IL-8**	7.5 (3.7-38.6)	34.0 (18.1-62.2)	0.095
**IL-18**	62.2 (34.0-101.9)	105.5 (63.8-274.3)	**0.027**
**MCP-1**	380.4 (202.7-467.5)	713.4 (554..7-964.5)	**0.015**
**PDGF-AA**	432.7 (229.3-691)	84.4 (70.8-143.1)	**0.01**
**PDGF-AB/BB**	4273 (2768.5-8974)	1231 (593.5-1984)	**0.002**
**RANTES**	3901 (2261-4813)	1258 (323.3-2813)	**0.037**

Cytokines pg/mL	Septic patients (n=25) at day 3		P value
	Survivors (n=14)	Non-survivors (n=11)	
**IL-6**	22.4 (8.7-60.2)	95.4 (15.8-288.5)	**0.028**
**IL-8**	7.3 (3.2-21.3)	27.5 (11.8-40.6)	**0.019**
**IL-18**	51.7 (31.5-125.1)	125.8 (82.0-288.5)	**0.008**
**MCP-1**	346.2 (233.4-485.0)	850.6 (370.9-917.2)	**0.025**
**M-CSF**	107.2 (55.3-310.5)	263.7 (148.7-546.9)	**0.049**
**PDGF-AA**	334.9 (225.7-663.6)	95.7 (74.4-411.5)	**0.017**
**PDGF-AB/BB**	5635 (2904.3-9908.3)	1294 (438.9-9086)	**0.033**
**RANTES**	3355.5 (920.6-4327.8)	1701 (176.9-4436)	0.239
